# Lenvatinib potentiates the antitumor efficacy of combined radiotherapy and PD-L1 blockade in lung adenocarcinoma

**DOI:** 10.1080/15384047.2025.2610526

**Published:** 2026-01-02

**Authors:** Yudi Liu, Ling Xiao, Xinyu Nie, Jiahua Lyu, Chengxi Tang, Linjie Li, Xue Zhang, Tao Li, Jianming Huang, Shichuan Zhang

**Affiliations:** a Department of Radiation, Sichuan Clinical Research Center for Cancer, Sichuan Cancer Hospital & Institute, Sichuan Cancer Center, School of Medicine, University of Electronic Science and Technology of China, Chengdu, People's Republic of China; b The People's Hospital of Kaizhou District, Chongqing, People's Republic of China; c Department of Radiation, Radiation Key Laboratory of Sichuan Province, Sichuan Clinical Research Center for Cancer, Sichuan Cancer Hospital & Institute, Sichuan Cancer Center, University of Electronic Science and Technology of China, Chengdu, People's Republic of China; d Biochemistry and Molecular Biology, Sichuan Clinical Research Center for Cancer, Sichuan Cancer Hospital & Institute, Sichuan Cancer Center, University of Electronic Science and Technology of China, Chengdu, People's Republic of China

**Keywords:** Lenvatinib, radiotherapy, PD-L1, VEGFR2, lung adenocarcinoma

## Abstract

**Background:**

The potential of Lenvatinib to synergize with combined radiotherapy and immunotherapy in LUAD remains incompletely characterized.

**Methods:**

We investigated Lenvatinib’s effects on radiation-induced PD-L1 in LUAD cells and VEGFR2 in HUVECs via Western blot, VEGFA expression via RT-qPCR/ELISA, and angiogenesis via immunofluorescence. LUAD-HUVEC crosstalk was modeled in vitro. In C57BL/6 mice bearing LUAD tumors, we evaluated the efficacy of RT and anti-PD-L1 with or without Lenvatinib, monitoring tumor growth, survival, and profiling the tumor microenvironment by mIHC and flow cytometry.

**Results:**

Lenvatinib suppressed radiation-induced PD-L1 and VEGFR2 expression, inhibited angiogenesis, and disrupted HUVEC-facilitated LUAD proliferation. The triple-combination (RT + anti-PD-L1 + Lenvatinib) significantly suppressed tumor progression (*P* < 0.05) and extended median survival (34 vs. 29.5 days, *P* < 0.05) versus dual therapy. It also enhanced intratumoral CD8^+^ T-cell infiltration and cytotoxicity, promoted M1-like macrophage polarization, and reduced regulatory T cell frequency and microvessel density.

**Conclusions:**

Lenvatinib potentiates RT and anti-PD-L1 therapy in LUAD through dual immune-vascular modulation, supporting the clinical translation of this triple-combination strategy.

## Graphical abstract

**Figure:**
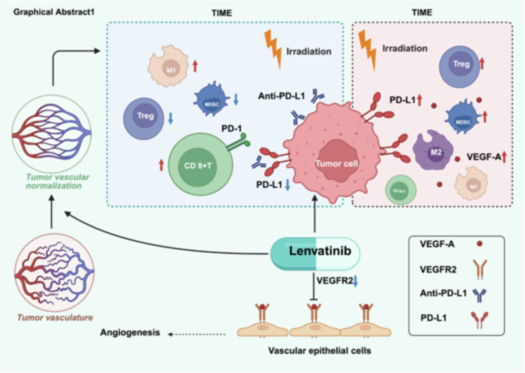

## Introduction

Lung cancer remains the most prevalent malignancy and leading cause of cancer-related mortality worldwide. For unresectable lung adenocarcinoma, radiotherapy (RT)—notably stereotactic body radiotherapy or stereotactic ablative radiotherapy (SBRT/SABR)—serves as a cornerstone treatment by combining direct tumour ablation with systemic immune activation via immunogenic cell death (ICD).^1-4^

Despite the success of RT in treating lung cancer, it cannot always completely eradicate the primary tumour, and local relapse and distant metastasis often occur followingirradiation. These limitations reflect the inability of RT-induced immune responses to establish durable systemic antitumor immunity. While immune checkpoint blockade has emerged as a transformative cancer treatment modality,^5-7^ its monotherapy efficacy remains constrained by low objective response rates (ORR, approximately 20%) in lung cancer,^8-10^ underscoring the need for combination strategies.

RT enhances immune checkpoint inhibitor (ICI) efficacy by promoting tumour antigen presentation and T-cell priming.^11^^,^^12^ However, this benefit is often limited by RT's “double-edged” effect: it concurrently induces compensatory immunosuppression through PD-L1 (Programmed Death-Ligand 1) upregulation and recruitment of myeloid-derived suppressor cells (MDSCs) and Regulatory T cells (Tregs).^13-15^ To overcome this, combining antiangiogenic (AA) agents with RT-ICI has emerged as a promising strategy, based on the rationale that AA-induced vascular normalisation can alleviate hypoxia and improve T-cell infiltration.^16-19^ However, the clinical translation of such dual combinations faces a critical translational barrier: spatio-temporal mismatch. The “vascular normalisation window” induced by AA agents is often transient and difficult to align with the delayed kinetics of RT-induced immune activation.^20-23^ This “asynchrony” highlights the need for an active “synchroniser” that can simultaneously target both RT-induced hypoxic resistance (vascular endothelial growth factor, VEGF) and adaptive immune resistance (PD-L1). Based on these complementary mechanisms, we hypothesise that a triple combination of RT, ICIs, and an antiangiogenic agent can remodel the immunosuppressive tumour microenvironment (TME) and achieve durable tumour control.

Lenvatinib, a multi-target tyrosine kinase inhibitor (TKI), mediates its antitumor effects through dual mechanisms of vascular normalisation via potent inhibition of vascular endothelial growth factor receptors 1-3 (VEGFR1-3), which reduces tumour hypoxia and improves perfusion, and immune microenvironment remodelling through enhanced CD8+ T cell infiltration and reduced Treg cell accumulation.^24^^,^^25^ Notably, unlike certain VEGF/VEGFR inhibitors (e.g., sorafenib, sunitinib, and bevacizumab) that primarily induce vessel pruning and potentially upregulate tumour PD-L1 expression, Lenvatinib demonstrates unique capabilities: it directly downregulates PD-L1 expression on tumour cells via fibroblast growth factor receptor (FGFR)-4 inhibition,^26^ causes immunomodulatory effects, including activation of effector T-cells and regulation of tumour-associated macrophages (TAMs), and establishes transient ‘vascular normalisation windows’. This dual effect enables simultaneous optimisation of radiotherapy dosimetry and T-cell infiltration.^27-30^ This tripartite synergy—simultaneous optimisation of radiation response and antitumor immunity—positions Lenvatinib as an ideal candidate for combination with radiotherapy and immunotherapy. Capitalising on these mechanistic foundations, our study systematically evaluates how lenvatinib-mediated TME reprogramming potentiates RT-ICI efficacy in lung adenocarcinoma (LUAD).

## Results

### Lenvatinib reduces radiation-induced PD-L1 expression in LUAD cells

**Figure 1.**
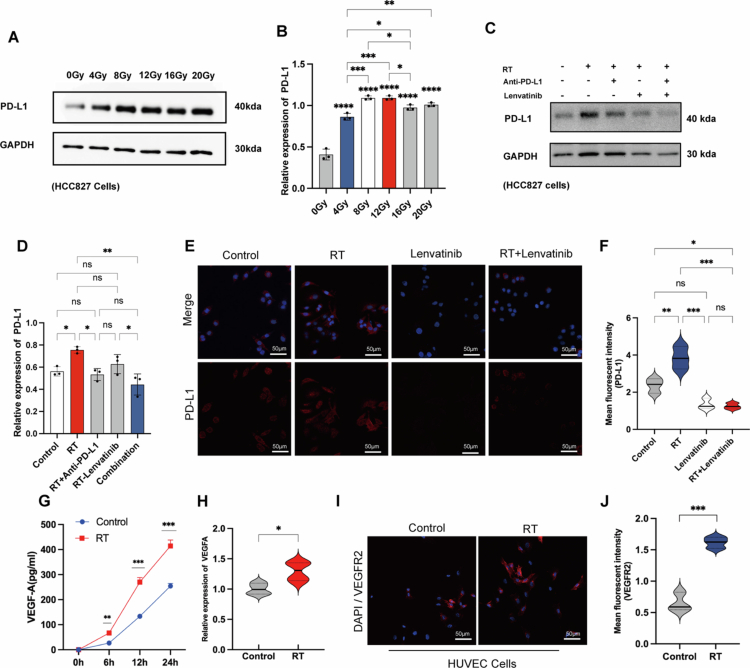
Radiation induces dual adaptive resistance pathways, and lenvatinib counteracts RT-induced PD-L1 upregulation. (A-B) Western blot analysis (A) and quantification (B) of PD-L1 protein expression in HCC827 cells 24 hours after gradient X-ray irradiation (0-20 Gy, *n* = 3). (C-D) Western blot analysis (C) and quantification (D) of PD-L1 expression in HCC827 cells 24 hours after 8 Gy irradiation, treated with lenvatinib (10 µM) and/or anti-PD-L1 antibody (40 µg/mL, *n* = 3). (E-F) Representative IF images (E) and quantification (F) of PD-L1 surface expression (MFI) in A549 cells treated with 8 Gy RT and Lenvatinib (10 µM, *n* = 3). Scale bar = 50 µm. (G) ELISA analysis of secreted VEGF-A protein in the supernatants of HCC827 cells at indicated time points (0-24 h) after 8 Gy irradiation (*n* = 3). (H) RT-qPCR analysis of *VEGFA* mRNA expression in HCC827 cells 24 hours after 8 Gy irradiation (*n* = 3). (I-J) Representative IF images (I) and quantification (J) of VEGFR2 expression in HUVECs (*n* = 3). Scale bar = 50 µm. Data are presented as mean ± SEM. Statistical analysis was performed using Student's t-test, Mann-Whitney U test, or One-way ANOVA. (ns: P ≥ 0.05, *: *P* < 0.05, **: *P* < 0.01, ***: *P* < 0.001, ****: *P* < 0.0001).

As shown in Figure 1A-B, Western blot analysis demonstrated that X-ray irradiation (0-20 Gy, 24 h) induced a dose-dependent upregulation of PD-L1 expression (*P* < 0.01). Consistent with this, in 8 Gy-irradiated HCC827 cells, treatment with lenvatinib, anti-PD-L1 antibody, or their combination significantly downregulated this radiation-induced PD-L1 expression compared to RT alone (*P* < 0.01; Figure 1C-D). Furthermore, immunofluorescence (IF) analysis confirmed that Lenvatinib treatment markedly reduced PD-L1 surface expression in A549 cells, as evidenced by a significant decrease in mean fluorescence intensity (MFI) compared to irradiated controls (*P* < 0.001; Figure 1E-F).

### Irradiation upregulates VEGF-A in LUAD cells and VEGFR2 in HUVECs

The VEGF-A/VEGFR2 signalling axis is a well-established mediator of pro-angiogenic crosstalk between tumour cells and endothelium. To investigate its activation by radiation in the context of LUAD, we employed orthogonal assays. In HCC827 cells, 8 Gy X-ray irradiation induced a time-dependent increase in VEGF-A, with both its mRNA (RT-qPCR) and secreted protein (ELISA) levels peaking at 24 hours post-irradiation (Figure 1G, H). Concurrently, 8 Gy irradiation also significantly upregulated the expression of its receptor (VEGFR2) in human umbilical vein endothelial cells (HUVECs), as confirmed by immunofluorescence (Figure 1I, J). Collectively, these data demonstrate that acute high-dose radiation rapidly establishes a paracrine VEGF-A/VEGFR2 signalling axis between LUAD and endothelial cells within 24 hours.

### Lenvatinib inhibits HUVEC proliferation mediated by LUAD cell-derived VEGF-A

**Figure 2.**
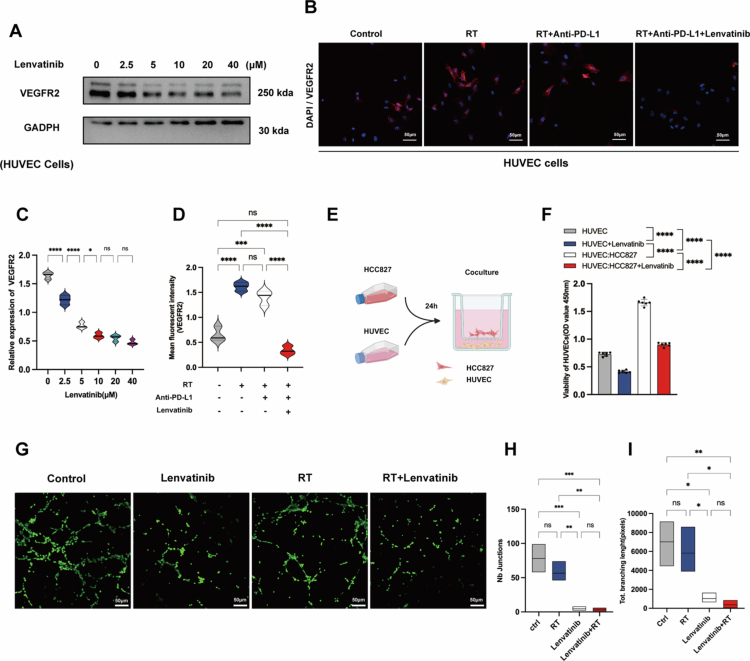
Lenvatinib inhibits tumour-induced endothelial activation via VEGFR2 blockade and angiogenic disruption. (A, C) Western blot analysis of VEGFR2 expression in HUVECs treated with increasing concentrations of lenvatinib (0-40 μM) for 24 hours (*n* = 3). (B, D) Representative IF images (B) and quantification (D) of VEGFR2 expression (MFI) in HUVECs treated with lenvatinib (10 µM, *n* = 3). Scale bar = 50 µm. (E) Schematic of the in vitro co-culture model, with HCC827 cells cultured on a 0.4-μm transwell insert above a monolayer of HUVECs (Created with BioRender.com). (F) CCK-8 viability assay of HUVECs cultured alone (monoculture) or co-cultured with HCC827 cells, with or without lenvatinib (10 µM) for 24 hours (*n* = 6). (G-I) In vitro tube formation assay using HUVECs cultured in conditioned medium from irradiated (8 Gy) HCC827 cells, which were pretreated with or without lenvatinib. (G) Representative images of Calcein AM-stained HUVEC networks (4-8 h). (H) Quantification of the number of junctions. (I) Quantification of total vascular length (*n* = 3). Scale bar = 50 µm. Data are presented as mean ± SEM. Statistics: Mann-Whitney U test or One-way ANOVA. (ns: P ≥ 0.05, *: *P* < 0.05, **: *P* < 0.01, ***: *P* < 0.001, ****: *P* < 0.0001).

VEGFA secreted by cancer cells activates VEGFR2 on vascular endothelial cells (VECs), driving endothelial proliferation, tumour angiogenesis, and LUAD progression.^31^^,^^32^ To evaluate the therapeutic potential of lenvatinib targeting VEGFR2 in counteracting LUAD-driven angiogenesis, we performed functional assays. Western blot and IF analyses demonstrated that lenvatinib significantly downregulated both the total abundance and activation of VEGFR2 in HUVECs compared to untreated controls (Figure 2A−D). Consequently, the CCK-8 assay confirmed that lenvatinib potently inhibited HUVEC proliferation in a co-culture system (*P* < 0.01; Figure 2E-F). These data establish that lenvatinib attenuates the pro-angiogenic capacity of LUAD cells through dual inhibition of the VEGF-A/VEGFR2 signalling axis.

### Lenvatinib reduces pro-angiogenic capacity of HUVEC induced by irradiated LUAD cells

To mechanistically interrogate lenvatinib’s anti-angiogenic efficacy in irradiated tumours, we conducted a standardised tube formation assay quantifying HUVEC network formation when cultured with conditioned medium from non-irradiated vs irradiated HCC827 cells with or without Lenvatinib pretreatment. Quantitative image analysis showed that lenvatinib prevented HUVECs from forming vascular lumens in a co-culture system in response to conditioned medium from irradiated cells (*P* < 0.01, Figure 2G-I) compared to the corresponding controls. These data demonstrate that Lenvatinib exerts dual anti-angiogenic effects by simultaneously suppressing VEGF-A production in LUAD cells and inhibiting VEGFR2 activation in endothelial cells, thereby effectively disrupting the VEGF-A/VEGFR2 signalling axis and reducing tumour angiogenesis.

### Lenvatinib remodels TME in LUAD-bearing mice

**Figure 3.**
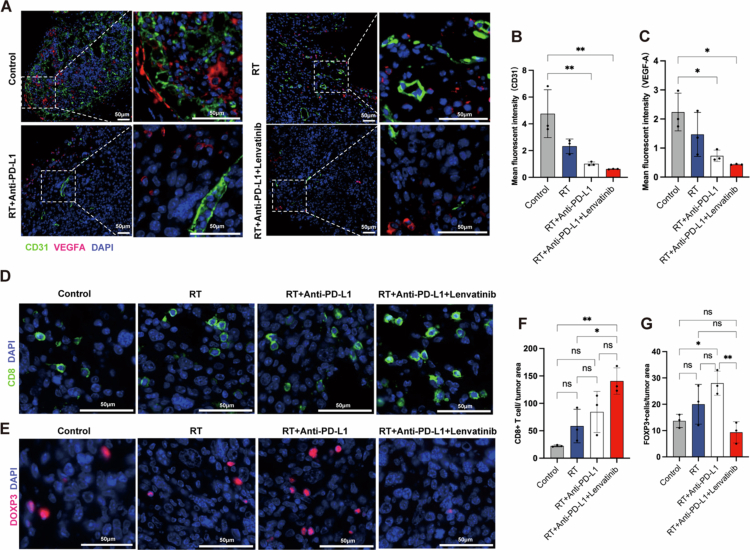
Triple therapy synergistically inhibits angiogenesis and reprograms the immunosuppressive TME. Tumour-bearing mice (LLC model) were randomised into four groups: Control, RT alone (8 Gy x 3), RT + anti-PD-L1, or Triple therapy (RT + anti-PD-L1 + lenvatinib). (A-C) mIHC analysis of tumour angiogenesis. (A) Representative images showing CD31 (green) for vasculature and VEGF-A (red). Nuclei are counterstained with DAPI (blue). (B) Quantification of CD31⁺ vascular density. (C) Quantification of VEGF-A fluorescence intensity (*n* = 3 mice/group). Scale bar: 50 μm. (D, F) IF analysis of CD8⁺ T-cell infiltration. (D) Representative images (CD8, green; DAPI, blue). (F) Quantification of CD8⁺ cell counts per field (*n* = 3 mice/group). Scale bar = 50 µm. (E, G) IF analysis of Treg infiltration. (E) Representative images (FOXP3, red; DAPI, blue). (G) Quantification of FOXP3⁺ cell counts per field (*n* = 3 mice/group). Scale bar = 50 µm. Data are presented as mean ± SEM. Statistics: One-way ANOVA with Tukey's *post hoc* test. (ns: P ≥ 0.05; *: *P* < 0.05; **: *P* < 0.01; ***: *P* < 0.001; ****: *P* < 0.0001).

**Figure 4.**
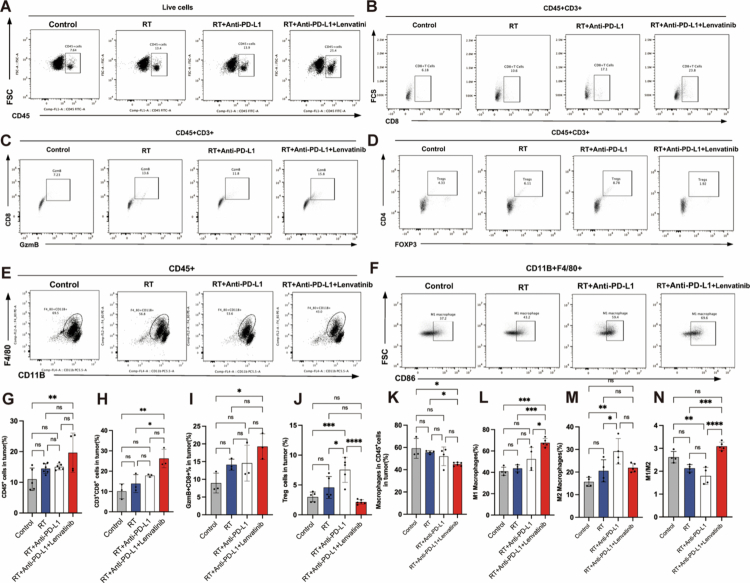
Triple therapy reprograms the immunosuppressive TME by activating cytotoxic immunity and repolarising macrophages. Flow cytometry analysis of dissociated tumours from the four treatment groups (Control, RT alone, RT + anti-PD-L1, Triple therapy; *n* = 3 mice/group). (A-F) Representative flow cytometry plots. (G-*N*) Quantification of: (G) total tumour-infiltrating CD45⁺ immune cells, (H) CD8⁺ T cells, (I) cytotoxic CD8⁺ T cells (Granzyme B⁺), (J) FOXP3⁺ Tregs, (K) total macrophages, (L) M1-polarised macrophages, (M) M2-polarised macrophages, and (*N*) the M1/M2 macrophage ratio. Data are presented as mean ± SEM. Statistics: One-way ANOVA with Tukey's *post hoc* test. (ns: P ≥ 0.05; *: *P* < 0.05; **: *P* < 0.01; ***: *P* < 0.001; ****: *P* < 0.0001).

Aberrant vasculature and immunosuppression within the TME jointly hinder RT/ICB efficacy.^33^^,^^34^ Vascular normalisation converts an immunosuppressive TME to an immune-permissive state, enhancing RT/ICB efficacy via improved T-cell infiltration.^35^ To determine whether lenvatinib induces vascular remodelling and immune phenotype modulation in the TME, we employed a Lewis lung carcinoma (LLC) model of LUAD. Quantitative analyses demonstrated that the triple-combination therapy (lenvatinib+ RT + anti-PD-L1) significantly reduced expression of the vascular endothelial marker (CD31+, *P* < 0.001), interstitial VEGF-A (*P* < 0.001) and microvascular density, compared to monotherapy or dual-combination groups (Figure 3A-C). In addition, lenvatinib combined with RT/anti-PD-L1 significantly increased the level of CD45+ cells and the ratio of M1/M2 macrophage ratio, fostered CD8+ T-cell infiltration and Gzm B secretion, and reduced the levels of MDSCs and Treg cells in the tumour tissues compared to RT or RT/anti-PD-L1 alone (Figure 3D-G, Figure 4, and Figure S1). Collectively, these data demonstrate that triple-combination therapy coordinately normalises radiation-induced pathological vasculature and favourably reprograms the immune landscape, thereby creating an immune-permissive TME.

### Lenvatinib enhances RT/anti-PD-L1 efficacy in LUAD-bearing mice

**Figure 5.**
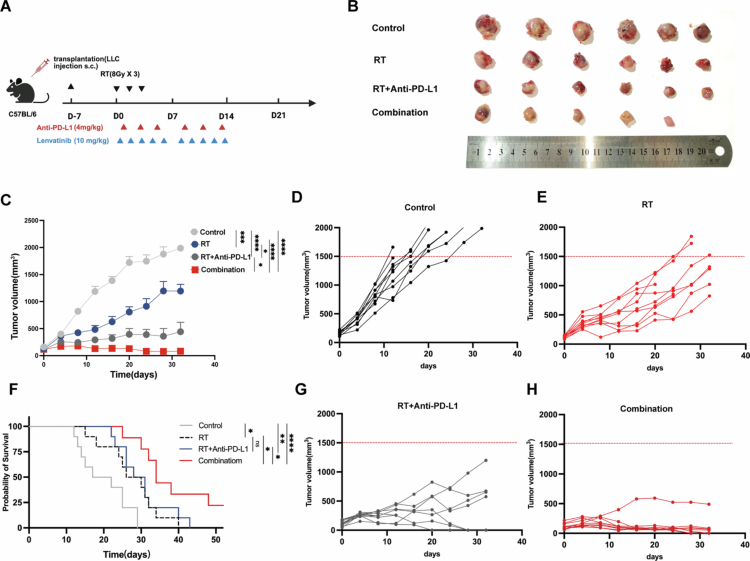
Triple therapy synergistically inhibits tumour growth and prolongs survival in LLC models. (A) Schematic of the in vivo treatment regimen (Created with BioRender.com). C57BL/6 mice bearing LLC tumours (*n* = 8/group) were randomised and treated as follows: Control (PBS); RT (8 Gy × 3 daily fractions); RT + anti-PD-L1 (10 mg/kg, i.p., 3 × /week for 2 weeks); Triple therapy (RT + anti-PD-L1 + lenvatinib, 4 mg/kg, p.o., 5 × /week for 2 weeks). (B) Representative images of excised tumours from each group at day 14 (*n* = 5~6/group). (C) Mean tumour growth curves for all four treatment groups (*n* = 7~10/group). (D, E, G, H) Individual tumour growth curves for each mouse in the (D) Control, (E) RT, (G) RT + anti-PD-L1, and (H) Triple therapy groups. (F) Kaplan-Meier survival analysis (*n* = 7~10/group). Survival cutoff was based on humane endpoints (tumour volume > 2000 mm³ ). Statistical analysis: Endpoint tumour volumes were analysed by one-way ANOVA with Tukey's post hoc test. Survival curves (F) were analysed by the Log-rank (Mantel-Cox) test. (ns: P ≥ 0.05; *: *P* < 0.05; **: *P* < 0.01; ***: *P* < 0.001; ****: *P* < 0.0001).

To evaluate the triple-combination therapy's synergistic antitumor effects, we used an LLC-bearing mouse model to comprehensively assess tumor growth and survival (Figure 5A). The combination therapy (RT + anti-PD-L1 + lenvatinib) significantly suppressed tumour growth and progression (Figure 5B-C) and prolonged median survival (Figure 5F). Quantitative analysis at day 24 post-treatment demonstrated significant therapeutic effects across groups, with mean tumour volumes of 1747 ± 235 mm³ (control), 913 ± 395 mm³ (RT alone, 47.7% reduction vs control, *P* < 0.001), 387 ± 277 mm³ (RT/anti-PD-L1, 77.8% reduction, *P* < 0.0001), and 125 ± 151 mm³ (triple therapy, 92.8% reduction, *P* < 0.0001). The triple-combination therapy demonstrated the strongest effect, achieving a further statistically significant reduction in tumour volume compared to the dual RT/anti-PD-L1 therapy (*P* < 0.05, Figure 5C-E, G, H). The Lenvatinib-containing triple-combination regimen showed superior survival outcomes with a median survival of 34 days compared to 29.5 days (RT/anti-PD-L1) (log-rank *P* < 0.05, Figure 5F). We next assessed the systemic toxicity and tolerability of the combination therapy. All treatment groups, including those receiving the triple-combination, maintained stable body weight throughout the study, with no significant weight loss observed (Figure S2B), indicating the regimen was well-tolerated. Furthermore, histopathological examination of major organs (heart, lungs, liver, and kidneys) via H&E staining at the endpoint revealed no evidence of treatment-related damage, inflammation, or pathological lesions in any group (Figure S2C). Collectively, our data demonstrate that lenvatinib potentiates the antitumor efficacy of the combined RT/anti-PD-L1 therapy while maintaining a favourable safety profile in a LUAD-bearing mouse model (Figure S2A-C).

## Discussion

Despite clinical evaluation of radiotherapy combined with ICIs or anti-angiogenic drugs, the efficacy of these dual therapies in advanced lung adenocarcinoma remains constrained.^36^ As neither approach alone can fully counteract the compensatory immunosuppressive and pro-angiogenic feedback in the TME, we reasoned that a more comprehensive strategy is needed. Thus, we propose that an orchestrated triple therapy, designed to co-activate immunity (RT), block immune checkpoints (ICI), and remodel the vasculature (anti-angiogenic agent), may be pivotal to overcoming resistance in LUAD.^23^^,^^27^^,^^37^ This pursuit of an optimal combination, however, is further complicated by the inherent challenge of finding an optimal sequence.

The critical yet inconsistent role of treatment sequencing highlights a fundamental paradox in dual-combination therapies. This is exemplified by recent conflicting evidence: where Sato et al. champion an anti-angiogenic-first approach, Lin et al. demonstrate superior outcomes with an ICB-first schedule.^38^^,^^39^ Such direct contradictions expose a core limitation: therapies inherently constrained by the need for precise “treatment sequence” possess a narrow and clinically unmanageable therapeutic window. Our study addresses this impasse by moving beyond sequential timing. We introduce a strategy of active synchronisation, wherein the multi-target agent lenvatinib concurrently orchestrates vascular normalisation and immune activation, thereby overcoming the temporal disconnect that plagues dual therapies.

High-dose radiation presents a similar therapeutic dilemma. While it can induce immunogenic cell death, it also drives compensatory resistance, notably the upregulation of PD-L1. We selected an 8 Gy dose because it induced peak PD-L1 expression *in vitro* (Figure 1A–B), a phenomenon potentially driven by synergistic transcriptional (cGAS-STING) and post-translational (ATR/CHK1) mechanisms.^40^^,^^41^ In this context, anti-PD-L1 antibody treatment effectively reversed the radiation-induced PD-L1 upregulation (Figure 1C, D), thereby blocking a major pathway of immune escape.^42^^,^^43^

We found that lenvatinib effectively counteracted high-dose radiation-induced PD-L1 upregulation (Figure 1C-F). This effect is potentially mediated by the FGFR-STAT1/3-PD-L1 pathway.^26^^,^^28^^,^^44^ The FGFR signalling pathway itself has been shown to drive PD-L1 transcription by activating downstream STAT1 and STAT3.^45^ Lenvatinib has been reported to target the FGFR4 axis, activating GSK3-*β* kinase, which subsequently promotes PD-L1 ubiquitination and proteasomal degradation.^26^ Furthermore, lenvatinib not only inhibited RT-induced PD-L1 upregulation but also directly counteracted hypoxia-driven angiogenesis. Indeed, our results showed that lenvatinib significantly downregulated VEGFR2 expression in HUVECs (Figure 2A-D) and effectively inhibited tube formation in assays using conditioned medium from irradiated LUAD cells (Figure 2G-I).

Crucially, the in vitro inhibition of PD-L1 and VEGFR2 also drove a fundamental remodelling of the in vivo tumour microenvironment. The lenvatinib-based triple therapy not only reduced VEGFA levels and CD31+ microvessel density (Figure 3A-C) but, more importantly, reversed the immunosuppressive state. The triple therapy significantly altered the immune landscape, increasing the M1/M2 macrophage ratio while reducing the infiltration of Tregs and MDSCs (Figures 3 and 4). We propose that lenvatinib fostered an immune-permissive microenvironment by blocking VEGF signalling and alleviating hypoxia. This remodelling promoted CD8+ T cell infiltration and cytotoxic function, as evidenced by increased Granzyme B secretion, effectively transforming immune “cold” tumours into “hot” tumours. Compared to all monotherapies and dual therapies, the triple therapy achieved significantly superior *in vivo* tumour control (Figure 5) without inducing additional systemic toxicity (Fig. S2A−C). This favourable safety and tolerability profile provides critical support for the potential clinical translation of this triple regimen.^30^^,^^46-48^

While this study provides mechanistic insights, several limitations warrant consideration. The generalisability of our conclusions, based on a single model system, must be tested in patient-derived xenograft or genetically engineered mouse models. Additionally, the static nature of our TME analysis could not capture the dynamic interplay of immune remodelling and did not focus on other key immune populations, such as DCs and NK cells.^49^ This area is particularly worthy of investigation, as RT in our regimen may actively mobilise these populations. RT has been shown to systemically induce Flt-3L, a key cytokine for DC mobilisation, while high intratumoral expression of its receptor, FLT3, is a favourable prognostic marker in NSCLC correlating with high DC/NK infiltration.^50^^,^^51^ Investigating how our lenvatinib-based triple therapy modulates this RT-induced Flt-3L/FLT3 axis is therefore a critical future direction. A more granular understanding of vascular normalisation, beyond CD31-based quantification, is also needed and could be achieved with CD31/α-SMA co-staining. Ultimately, translating these findings will require identifying predictive biomarkers, such as FGFR status or baseline hypoxia,^52^ to select patients most likely to benefit.

In summary, this study establishes a triple therapy that generates a self-reinforcing anti-tumour immune cycle. High-dose radiation initiates ICD, and anti-PD-L1 blocks adaptive immune resistance. By concurrently inhibiting FGFR (to target PD-L1) and VEGFR2 (to counteract hypoxia), lenvatinib not only mitigates RT-induced resistance but also actively remodels the TME—polarising macrophages toward an M1 phenotype and reducing Tregs/MDSCs—to enhance T-cell cytotoxicity. This coordinated strategy overcomes the “spatiotemporal mismatch” that plagues dual-combination therapies, providing a robust preclinical rationale and a strategic framework for future clinical trials in LUAD.

## Conclusion

Lenvatinib potentiates the antitumor efficacy of combined RT and anti-PD-L1 therapy in LUAD through dual modulation of immune and vascular compartments. This work provides a preclinical foundation for its clinical translation as a radio-immunotherapy combination partner, pending further validation.

## Materials and methods

### Cell lines and cultures

Lewis lung carcinoma (LLC), A549, and HCC827 cells were obtained from ATCC; HUVECs were provided by Sichuan Cancer Hospital. LLC and A549 cells were cultured in high-glucose DMEM (Gibco), HCC827 cells in RPMI-1640 (Gibco), and HUVECs in endothelial cell medium (ScienCell #1001). All media were supplemented with 10% FBS and 1% penicillin-streptomycin (Thermo Fisher), and cells were maintained at 37 °C with 5% CO₂.

### LLC-bearing mice model

The experimental unit was the individual animal. A total of 34 female C57BL/6 mice (6–8 weeks old, 18–20 g; Chongqing Tengxin Biotechnology) were housed under SPF conditions. For subcutaneous tumour modelling, 1 × 10⁶ LLC cells suspended in 100 μL PBS were inoculated subcutaneously into the right lower flank of each mouse. On Day 7 post-inoculation, when tumours reached 100–200 mm³, 32 tumour-bearing mice were randomised into four experimental groups (*n* = 8 per group). Mice were randomised using a stratified method based on tumour volume, with an allocation sequence generated by a computer-based random number generator. To ensure allocation concealment, an independent investigator not involved in the study generated the sequence and assigned animals, revealing the allocation only after enrolment. Two mice that did not develop tumours were excluded prior to randomisation. Tumour volume was measured every 3 days using a vernier caliper (Volume = 0.5 × length × width²) by a single blinded investigator. Humane endpoints (tumour volume >2000 mm³ or >20% body weight loss for 48 consecutive hours) triggered euthanasia via sodium pentobarbital (100 mg/kg, i.p.) followed by cervical dislocation. All procedures were approved by Sichuan Cancer Hospital's Ethics Committee (No. SCCEC-04-2023-011) and complied with ARRIVE guidelines.

### Drug administration and irradiation in LLC-bearing mice

LLC-bearing mice received oral lenvatinib (4 mg/kg, 5 times per week for 2 weeks; Selleck #S1164). The dosage regimen was selected based on previous studies that had confirmed the antitumor efficacy and acceptable safety profile of this agent.^53^ Anti-PD-L1 antibody (10 mg/kg, 3 times per week for 2 weeks; Bio X Cell) was administered intraperitoneally in InVivoPure diluent (#IP0070), while controls received PBS. For irradiation, anaesthetised mice (1% pentobarbital) were immobilised in an X-Rad320 irradiator. Tumours received 8 Gy/fraction × 3 daily fractions (160 kV, 1.922 Gy/min) with lead-shielded normal tissues. This specific fractionation schedule was selected as it has been extensively validated to optimally induce immunogenic cell death (ICD) and prime downstream systemic antitumor immunity, unlike single high-dose regimens.^54^^,^^55^ Survival (time to euthanasia/death) was analysed by Kaplan-Meier method (GraphPad Prism).

### Flow cytometric analysis of tumour-infiltrating immune cells

Tumour tissues from LLC-bearing mice were dissociated with collagenase I and DNase I (Solarbio), filtered through a 70-μm strainer (Falcon, #352350), and treated with RBC lysis buffer. For flow cytometry, cells were stained with APC/Cy7 Fixable Viability Dye 780 (BD Biosciences, #565388) for live/dead discrimination, followed by Fc block with anti-CD16/32 (BD Biosciences, #553083). The following antibody panel was employed for surface and intracellular staining: T lymphocytes: FITC-anti-CD45 (553079), PE-Cy7-anti-CD3ε (552774), APC-anti-CD4 (553051), PerCP-Cy5.5-anti-CD8α (551162); Regulatory T cells: PE-anti-Foxp3 (563101); Cytotoxic markers: Alexa Fluor 647-anti-granzyme B (515406); Myeloid cells: PE-anti-F4/80 (565410), PE-Cy7-anti-CD86 (560582), Alexa 647-anti-CD206 (565250), PerCP-Cy5.5-anti-CD11b (550993); Granulocytes: APC-anti-Ly-6G (560599), PE-Cy7-anti-Ly-6C (560593). Intracellular staining employed BD Transcription Factor Buffer Set (#0325663), with compensation via UltraComp eBeads (Thermo Fisher, #01-2222-42). Samples were acquired on a BD FACSCanto II and analysed in FlowJo v10.9.0 (gating strategy: Supplementary Figures S3-S4).

### Histopathological and multiplex immunohistochemical analysis

Tumour samples were fixed in 4% paraformaldehyde, dehydrated through graded ethanol, and embedded in paraffin. Sections (4 μm) were stained with hematoxylin and eosin (H&E; Solarbio, G1121) for histopathology. Bright-field images of five random fields per sample were captured (OLYMPUS BX53 microscope).

For multiplex immunohistochemistry (mIHC), deparaffinised sections underwent microwave antigen retrieval in Tris-EDTA buffer (pH 9.0), followed by endogenous peroxidase blockade (3% H₂O₂, 10 min) and blocking with 10% goat serum (30 min). Primary antibodies were incubated overnight at 4 °C: CD8α (1:4,000; abcam, ab209775), CD31 (1:25,000; abcam, ab182981), VEGFA (1:200; Proteintech, 19003-1-AP), FoxP3 (1:1,000; Cell Signalling Technology, #12653). Multiplex detection used a four-colour kit (Absin, abs50028-20T) per manufacturer's protocol. High-resolution confocal images (3-4 random fields/section) were acquired (Nikon A1R SI). Quantitative analysis employed ImageJ with threshold-based cell counting.

### Cell co-culture and treatment conditions

HUVECs (1 × 10⁵ cells/cm²) and HCC827 cells (1 × 10⁵ cells/cm²) were co-cultured using 0.4 µm pore inserts (Corning, #353493) in 6-well plates. Cells were maintained in a 1:1 mixture of endothelial complete medium (ScienCell) and RPMI-1640 (Gibco) with 10% FBS. After exposure to 8 Gy X-rays (X-RAD 320, 160 kV, 25 mA; 1.922 Gy/min), cells were treated with 10 µM lenvatinib (Selleckchem, #S1164) and 40 µg/mL anti-PD-L1 mAb (Selleckchem, #A2004) for 24 h.

### CCK-8 assay

To assess the combinatorial effects of anti-PD-L1 and lenvatinib on proliferation, co-cultured HCC827 and HUVECs (5 × 10³ cells/well) were seeded in 96-well plates. After 24 h, 10 µL CCK-8 reagent (MCE, #HY-K0301) was added per well and incubated for 2 h at 37 °C. Absorbance at 450 nm was measured using a microplate reader. Experiments were performed in triplicate. Cell viability (%) was calculated as: [(OD sample - OD blank)/(OD control - OD blank)] × 100%.

### In vitro angiogenesis assay

Growth factor-reduced Matrigel (Corning, #354263) was diluted to 10 mg/mL in serum-free medium. Pre-chilled 24-well plates were coated with 300 µL/well and gelled at 37 °C for 30 min. HUVECs (1 × 10⁵ cells/well) from treatment groups were seeded onto the gel in complete medium. Tube formation was monitored hourly (2-12 h) by phase-contrast microscopy (Olympus IX83). For fluorescence, cells were stained with 2 µM Calcein AM (Beyotime, #C2012; 30 min, 37 °C, dark). Three random fields/well were imaged (Nikon A1R SI). Tube formation was quantified using ImageJ Angiogenesis Analyser.

### RT-qPCR

Total RNA was extracted with Trizol (Invitrogen, #15596026CN). cDNA was synthesised using ExonScript RT SuperMix with dsDNase (EXONGEN, #A502-02). PCR reactions were performed using PowerUp SYBR Green Master Mix (Thermo Fisher, #A25742). The gene-specific primers used were as follows: Human VEGFA forward, 5’-AGGGCAGAATCATCACGAAGT-3’, reverse, 5’-AGGGTCTC GATTGGATGGCA-3’. Human *β*-Actin forward, 5’-GCACAGAGCCTCGCCTTT-3’, reverse, 5’-CACAGGACTCCATGCCCAG-3’. RT-qPCR was run on a CFX96 Touch system. Gene expression was normalised to *β*-Actin and analysed via the 2^(−ΔΔCt) method.

### Western blot

Total protein was extracted with RIPA buffer (Solarbio, #R0010) containing protease/phosphatase inhibitors (Beyotime, #P1045), and concentrations were determined by BCA assay (Solarbio, #PC0020). Proteins (40 µg) were separated via SDS-PAGE, transferred to PVDF membranes, blocked with 5% skim milk in TBST (0.05% Tween-20) for one hour at RT, and incubated overnight at 4 °C with primary antibodies: VEGFR2 (D5B1 Rabbit mAb, 1:1,000, CST #9698), PD-L1 (1:1,000, Abcam #ab213524), and GAPDH (D16H11 XP® Rabbit mAb, 1:1,000, CST #5174). Membranes were then incubated with HRP-linked Anti-rabbit secondary antibody (1:10,000, CST #7074, 1 h, RT), detected using an ECL kit (Oriscience, #PD202), and analysed on a Tanon 4200 system.

### ELISA

VEGF-A protein in tumour cell supernatants was measured using an ELISA kit (Elabscience #E-EL-H0111) following the manufacturer's protocol, with absorbance at 450 nm. Relative levels were derived by normalising VEGF-A concentrations to total protein content per sample.

### Statistical analysis

Statistical analyses were performed using GraphPad Prism 9.0 (GraphPad Software). After confirming normality via Shapiro-Wilk test (*P* > 0.05), normally distributed data were expressed as mean ± SD. Group comparisons included: unpaired t-test (two groups), one-way ANOVA with Tukey’s *post hoc* (multi-group), Mann-Whitney U test (non-Gaussian two groups), and Kruskal-Wallis test with Dunn’s correction (non-Gaussian multi-group). Survival rates were analysed by Kaplan-Meier method with log-rank test, with *P* < 0.05 defining statistical significance.

## Acknowledgments

Not applicable.

## Author contributions

Yudi Liu: Writing- Original draft preparation, Investigation, Data curation. Ling Xiao: Methodology, Investigation, Formal analysis, Data curation. Xinyu Nie: Methodology, Investigation. Jiahua Lyu: Methodology, Conceptualisation. Chengxi Tang: Methodology, Conceptualisation. Linjie Li: Methodology, Investigation. Xue Zhang: Methodology, Investigation. Tao Li: Funding acquisition, Methodology, Conceptualisation. Jianming Huang: Project administration, Writing–review & editing, Conceptualisation. Shichuan Zhang: Project administration, Writing—review & editing, Conceptualisation.

## Supplementary Material

Supplementary material.docx Supplementary material.docx

## Data Availability

The raw data in this study may be obtained from the corresponding author upon reasonable request.

## Abbreviations

AA: Antiangiogenic
ATCC: American Type Culture Collection
ELISA: Enzyme-linked immunosorbent assay
FGFR: Fibroblast growth factor receptor
GzmB: Granzyme B
HUVECs: Human umbilical vein endothelial cells
ICD: Immunogenic cell death
ICI: Immune checkpoint inhibitors
IF: Immunofluorescence
LLC: Lewis lung carcinoma
LUAD: Lung adenocarcinoma
MDSC: Myeloid-derived suppressor cell
mIHC: Multiplex immunohistochemistry
ORR: Objective Response Rate
PD-L1: Programmed Death-Ligand 1
RT: Radiotherapy
SBRT: Stereotactic body radiation therapy
SABR: Stereotactic ablative radiotherapy
TAMs: Tumour-Associated Macrophages
TKI: Tyrosine Kinase Inhibitor
TME: Tumour microenvironment
Tregs: Regulatory T cells
VECs: Vascular endothelial cells
VEGF: Vascular endothelial growth factor
