# Supplementary material for: Lenvatinib potentiates the antitumor efficacy of combined radiotherapy and PD-L1 blockade in lung adenocarcinoma
Source: Cancer Biol Ther. 2026 Jan 2;27(1):2610526. doi: 10.1080/15384047.2025.2610526 (PMC12773621; doi:10.1080/15384047.2025.2610526)
Supplement: Supplementary material.docx [file KCBT_A_2610526_SM6036.docx]

**Supplementary material**

**Figure 1. Combination therapy reverses the immunosuppressive microenvironment by co-targeting M2-TAMs and MDSC subsets.**

**(A)** Representative Flow cytometry plots showing the proportion of M2 macrophages (CD11B⁺F4/80⁺CD206⁺) in tumor tissues among treatment groups. **(B)** Representative Flow cytometry gating strategies for M-MDSCs (CD11B⁺Ly6C⁺) and PMN-MDSCs (CD11B⁺Ly6G⁺) in tumor tissues. **(C)** Quantitative analysis of M2 macrophage infiltration (%) in tumor tissues. **(D)** Quantitative analysis of M-MDSC (CD11B⁺Ly6C⁺) infiltration (%) in tumor tissues. **(E)** Quantitative analysis of PMN-MDSC (CD11B⁺Ly6G⁺) infiltration (%) in tumor tissues. Data are presented as mean ± SEM. Significance levels: *p < 0.05, **p < 0.01, ***p < 0.001, ****p < 0.0001; ns, not significant. Statistical analysis was performed using one-way ANOVA with appropriate post-hoc tests.

**
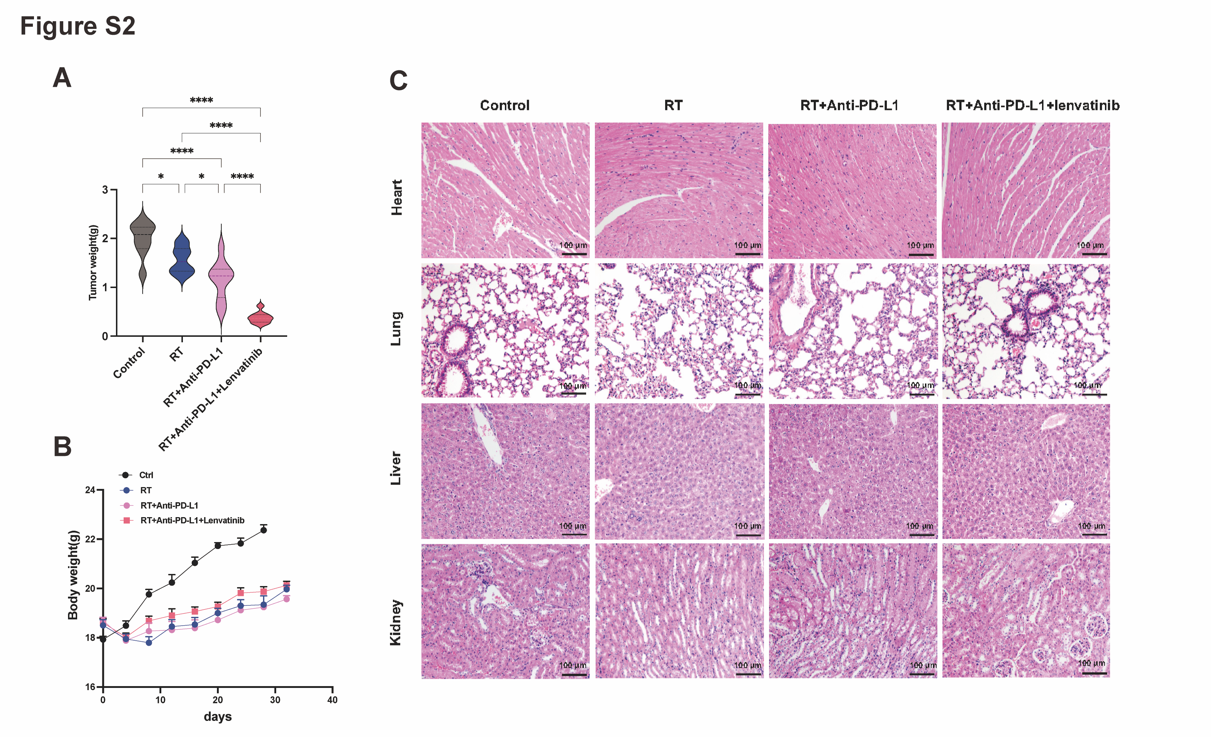
**

**Figure 2. Systemic safety profile and antitumor efficacy of combination therapy in xenograft models.**

**(A)** Body weight changes in tumor-bearing mice among different treatment groups. Measurements were recorded every three days starting from day 30 post-xenograft inoculation (n = 10). **(B)**Tumor weight comparison among groups. Tumors were harvested and weighed at the experimental endpoint (n = 10 per group). **(C)** Representative H&E-stained sections of heart, lung, liver, and kidney tissues from each treatment group. Scale bar: 100 μm. Data are expressed as mean ± SEM. Statistical significance: *p < 0.05, **p < 0.01, ***p < 0.001, ****p < 0.0001.

**Figure 3. Flow cytometry gating strategy for CD8+ T cells and TAMs in mouse tumors.**
**(A-B)** Representative Flow cytometry gating hierarchy: Live CD45+ cells were sequentially gated to identify CD3+ T cells (CD8+ T cells, GzmB+ CD8+ T cells), tumor-associated macrophages (TAMs; M1-Mϕ and M2-Mϕ subsets).

**Figure 4. Flow cytometry gating strategy for Treg cells and MDSCs in mouse tumors.**

**(A-B)** Representative Flow cytometry gating hierarchy: Live CD45+ cells were sequentially gated to identify CD3+ T cells (CD4+T cells, and regulatory T cells [Tregs]), polymorphonuclear MDSCs (PMN-MDSCs), and monocytic MDSCs (M-MDSCs)
